# Primary Care Practice Factors Associated With Telehealth Adoption in the United States: Cross-Sectional Survey Analysis

**DOI:** 10.2196/70404

**Published:** 2025-03-28

**Authors:** Matthew Mackwood, Elliott Fisher, Rachel O Schmidt, A James O'Malley, Hector P Rodriguez, Stephen Shortell, Ellesse-Roselee Akré, Alena Berube, Karen E Schifferdecker

**Affiliations:** 1 Department of Community & Family Medicine Geisel School of Medicine Dartmouth College Hanover, NH United States; 2 The Dartmouth Institute for Health Policy & Clinical Practice Geisel School of Medicine Dartmouth College Hanover, NH United States; 3 Department of Medicine Geisel School of Medicine Dartmouth College Hanover, NH United States; 4 Department of Biomedical Data Science Geisel School of Medicine Dartmouth College Hanover, NH United States; 5 School of Public Health University of California, Berkeley Berkeley, CA United States; 6 Department of Health Policy and Management Bloomberg School of Public Health Johns Hopkins University Baltimore, MD United States

**Keywords:** telehealth, telemedicine, remote consultation, primary health care, general practice, internet access, health policy, health care economics and organizations, access to primary care, digital divide, vulnerable populations, medically underserved area

## Abstract

In this national study of primary care practice–level factors associated with telehealth adoption in 2022, we found that training and assisting patients with the use of telehealth, broadband expansion efforts, and a higher proportion of low-income patients were associated with higher practice-level telehealth use, suggesting both opportunities for telehealth expansion and potential populations with higher need for its use.

## Introduction

In 2021, the National Academy of Medicine called for further study of telehealth to help strengthen primary care as part of their “Implementing High-Quality Primary Care in the United States” report [1]. Past studies highlight a range of patterns in telehealth use by patient-level factors [2], but primary care practice–level capabilities, such as resources to enable telehealth, have not been systematically assessed. Organizational and contextual factors, such as a federally qualified health center (FQHC) designation and a practice’s neighborhood characteristics, may also be associated with telehealth use. We analyzed a national survey of US adult primary care practices to examine relationships between practice-level factors and telehealth adoption.

## Methods

### Overview

We analyzed cross-sectional data from the 2022-2023 National Survey of Healthcare Organizations and Systems, which surveyed practice leaders about telehealth use in 2021, practice payment models, and care delivery structures and processes (1245/3499 people responded to the survey, for a 36% response rate). Survey administration information and data sources are described in Multimedia Appendix 1.

The primary outcomes are the proportion of all outpatient visits done via telehealth (real-time audiovisual or audio-only, eg, telephone) and the proportion of telehealth visits done audio-only. We categorized practices by quartile of telehealth use to compare practice-level differences by characteristic. We estimated multivariable linear regression models for all cases (N=1071) and used average marginal effects to generate estimates and 95% CIs for the primary outcome measures. Model covariates included practice ownership, clinician staffing, FQHC status, and telehealth-enabling resources. All analyses were completed in Stata (version 17; StataCorp), and all models used robust estimates of variance and 2-tailed *P* values, with *P*<.05 set as the threshold for significance. Weights were used in all analyses to account for nonresponse probabilities, detailed in Multimedia Appendix 1.

### Ethical Considerations

This study was deemed exempt by the Dartmouth College Committee for the Protection of Human Subjects (00032337). As practices were the unit of analysis, participants received an information sheet in lieu of informed consent. Practices were deidentified prior to analysis.

## Results

The median use of telehealth for practice encounters was 20% (IQR 10%-35%), and the median proportion of audio-only telehealth visits was 29% (IQR 17%-50%). High-telehealth-use practices were more likely to care for a high proportion of uninsured patients, to have expanded broadband access for patients, and to have trained patients to use telehealth (Table 1).

**Figure 1:**
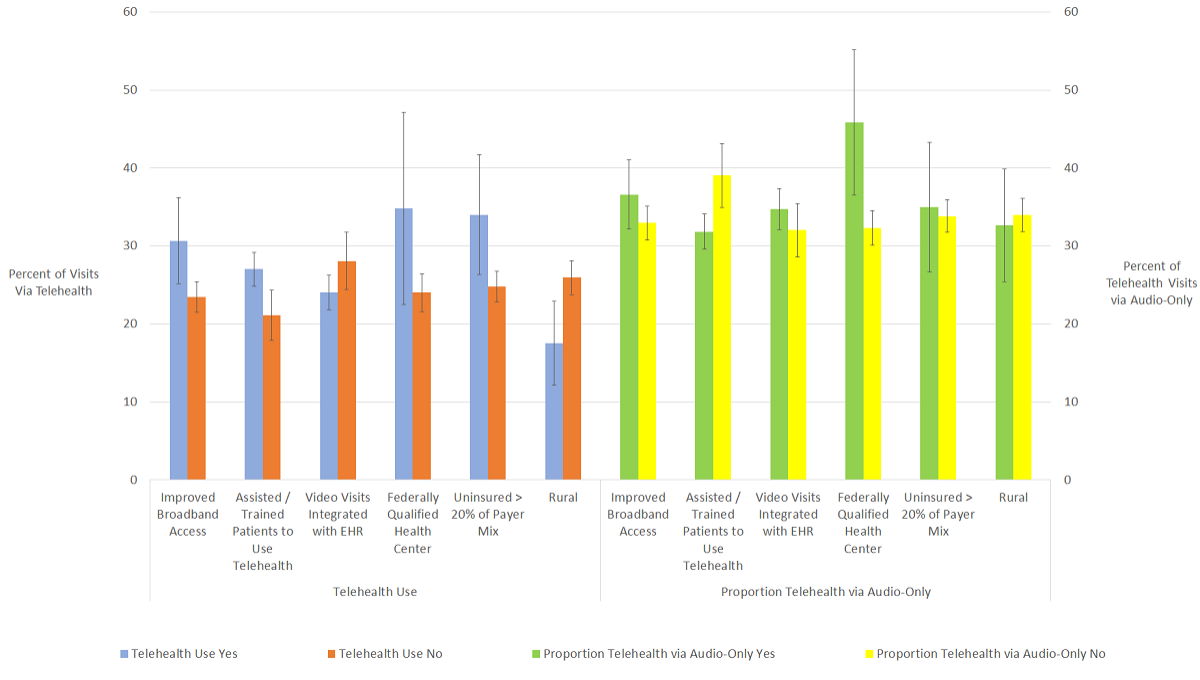
Adjusted Telehealth Use Rates and Audio-Only Telehealth Proportions by Select Factors. Covariates included in the multivariable linear regression model were as follows: practice ownership type, US census region, clinician staffing size, Area Deprivation Index quartile, median download speed at the census tract level, reported financial health, whether a practice reported impacts from staff shortages, alternative (to fee-for-service) payment model participation, and a payer mix that was >20% Medicare. Each covariate was significant in univariate regression at the *P*<.1 level; in preliminary runs when 2 factors had a Spearman correlation coefficient >0.5 or <–0.5, the least significant factor was excluded. EHR: electronic health record.

In multivariable regression analyses (Figure 1), a high payer mix of uninsured patients and high broadband expansion corresponded to higher telehealth use (*P*=.02 and *P*=.008, respectively), while rurality corresponded to lower telehealth use (*P*=.008). Audio-only telehealth use was higher among FQHCs compared to other practices (*P*=.02), and assisting patients with using telehealth was associated with both higher telehealth use and a smaller proportion of audio-only telehealth use (*P*=.002 and *P*=.001, respectively).

**Table 1:** Practice demographics (N=1071), overall and stratified by telehealth use quartile.

Characteristics^a^			Overall, %	Practice quartile for telehealth use, %					*P* value^b^	
				Lowest (0%-10%)	Second (>10%-20%)	Third (>20%-35%)	Highest (>35%)			
**Practice structure**										
	**Ownership**									.53
		Independent	25.4	27.9	27.4	24.6	21.2			
		Physician group	7.1	5.6	9.5	9.8	4.1			
		Hospital	15.7	18.8	15.4	9.4	17.2			
		Health system	37.9	38.5	37.4	40.3	39.9			
		Federally qualified health center or look-alike	13.6	8.9	10	15.6	17.4			
	**Physician count, n**									.05
		0-4	39.5	37.9	46.7	27.4	43.9			
		5-9	37	32	37.4	52	30.3			
		10-19	12.6	13.8	9.7	9.6	16.7			
		>20	10.9	16.3	6.2	11	9.1			
	**Advanced practice provider (physician assistant, advanced registered practice nurse) count, n**									.04
		Zero	21.4	28.8	24.7	7.5	20.3			
		1 or 2	27.2	19.7	34.1	33.8	23.7			
		3 or 4	23.9	20.8	21.1	25	29.8			
		5 to 10	18	17	12.7	24.6	19.5			
		>10 ()	9.5%	13.7	7.5	9.1	6.6			
**Practice financial characteristics**										
		Self-reported poor financial health	8.7	8.2	6.6	10.5	10.3	.80		
		Current alternative payment model participation^c^	81.5	78.1	86	83.6	79.5	.51		
		Impacted by physician workforce shortages	56.4	49.9	55.1	57.9	64.5	.41		
		Impacted by staff shortages	74	68.4	73.1	77.8	78.5	.33		
	**Payer mix (>20% of revenue from listed sources)**									
		Commercial	84.1	86.3	84.1	80.9	84.2	.68		
		Medicare	80.3	88.8	74.4	76.1	79.5	.10		
		Medicaid	34.1	31.8	33.4	35.1	36.7	.94		
		Uninsured	6.2	2.9	6	8.5	8.6	.04		
**Telehealth enablement factors**										
	Facilitated telehealth for patients by improving broadband access		27.7	19.7	24.7	25.7	42.6	.02		
	Facilitated telehealth by assisting or training patients to use telehealth		70.5	52.6	75.7	74.3	84.5	<.001		
	Platform for video visit integrated with electronic health record		69.4	70.2	69.3	68.1	69.7	.99		
**Census tract–level indicators**										
	Practice in a rural location		7.4	11.6	6.1	7.5	3.6	.10		
	**Area Deprivation Index quartile**									.40
		Most deprived	26.5	18.1	27	24.7	37.9			
		2nd quartile	(32.9	38.8	29.5	35.8	26.5			
		3rd quartile	22.9	24.6	25.3	22.2	18.9			
		Least deprived	17.8	18.6	18.2	17.3	16.7			
	**Internet speed measures (Mbps), mean (SD)**									
		Median download speed	76 (1.5)	70 (2.5)	78 (3.3)	73 (3.9)	83 (3.5)	.19		
		Median upload speed	16 (0.9)	15 (1.0)	17 (1.7)	14 (1.2)	18 (2.9)	.39		

^a^Characteristics are reported as weighted percentages unless otherwise noted. Details on weighting are provided in Multimedia Appendix 1.

^b^Differences between quartiles; generated via the *χ*^2^ test with the exception of download and upload speed, which were generated with the adjusted Wald test.

^c^Includes any engagement in accountable care organization and capitated payment contracts, which are alternatives to fee-for-service billing.

## Discussion

In this national study of primary care practices in 2022, we found that respondent practices serving the uninsured reported greater use of telehealth for patient encounters, and FQHCs used more audio-only telehealth. This suggests that low socioeconomic status populations had a higher need for telehealth and that cuts to audio-only reimbursement would disproportionately impact care for such patients. The “digital divide,” systematic barriers for accessing and using technology and telehealth among various populations [3], is a known issue for FQHC and rural populations [4,5], and our study provides national evidence that these practice-level factors are significantly associated with lower adoption of video-based telehealth.

Importantly, we found that the telehealth-enabling practices of training and assisting patients with using telehealth and broadband expansion were associated with higher telehealth adoption. In contrast, electronic health record integration for video visits, participation in alternative payment models that incentivize care quality, and practice ownership were not significant practice-level factors, contrasting with a prior study suggesting health system integration was linked to higher telehealth use at the physician level [6].

Limitations include potential nonresponse bias due to the modest response rate, though we used weights to account for this (sensitivity analyses of missing data are in Multimedia Appendix 1); that telehealth use is reported in aggregate rather than calculated from visit data, so could not be verified; and an inability to draw causal inference from cross-sectional analysis. Longitudinal analyses or controlled trials would provide stronger evidence and be able to describe any changes in significance of the identified factors for telehealth use over time.

These findings provide important national data for the design of policy and practice interventions to expand telehealth use. Practices focused on enabling telehealth appear able to meaningfully increase its uptake [7]. Federally, renewing the lapsed support for broadband accessibility is an important means to address the digital divide [8,9].

## Appendix

Multimedia Appendix 1 Further information on survey administration, analytic datasets, weights, and missing data.

## Abbreviations

APRN: advanced registered practice nurse
FQHC: federally qualified health center
PA: physician assistant

## Data Availability

The datasets generated or analyzed during this study are not publicly available due to the need for curating deidentified data, but are available on reasonable request from KES (karen.e.schifferdecker@dartmouth.edu).

## Disclaimer

The statements, findings, conclusions, views, and opinions contained and expressed in this article are based in part on data obtained under license from IQVIA information services (OneKey subscription information services 2017-2022, IQVIA Inc). The statements, findings, conclusions, views, and opinions contained and expressed herein are not necessarily those of IQVIA Inc or any of its affiliated or subsidiary entities.
