# Supplementary material for: Primary Care Practice Factors Associated With Telehealth Adoption in the United States: Cross-Sectional Survey Analysis
Source: J Med Internet Res. 2025 Mar 28;27:e70404. doi: 10.2196/70404 (PMC11992486; doi:10.2196/70404)
Supplement: Multimedia Appendix 1 [file jmir_v27i1e70404_app1.docx]

## Further Information on Survey Administration, Analytic Datasets, Weights, and Missing Data

The National Survey of Healthcare Organizations and Systems second wave (NSHOS Wave 2) was administered in 2022-2023; administration is described in detail elsewhere[1]. In brief, we contracted the market research firm SSRS, to proceed through a succession of methods to contact primary care practice leaders at a sample of 3,499 practices to administer a 52-item questionnaire. Surveys were administered from June 2022 through February 2023. Surveys which were > 50% incomplete were excluded. For this analysis, in addition to the NSHOS 2 responses, we augmented the data with additional measures about each practice at the Census tract level including the Area Deprivation Index (ADI), a global measure of socio-economic disadvantage[2]; rurality, defined using rural-urban commuting area codes[3]; practice ownership and clinician staffing from IQVIA’s 2022 OneKey data[4]; and 2020 Ookla download & upload speed data[5].

Details on survey weights and a comparison of the survey’s respondents to non-respondents are included in the supplemental materials of a prior publication[1]. Briefly, survey weights reflected the probability of a practice’s inclusion in the original Wave 1 (2017-2018) study sample or from the 2022 practice population sampling frame, as well as whether the practice responded to the survey (to account for non-response), in order to allow the sample analyzed to correspond to the sample frame based on the 2022 population of medical practices in the US.

In terms of missing data, of the 1,245 survey respondents, a total of 174 (14%) did not answer any questions about telehealth use; these practices were similar to those without missing data in all factors examined in table 1 except for being less likely to have > 20% of revenue from commercial insurance (67% vs 84%, p=.002), more likely to have > 20% of revenue from uninsured, and more likely to be an FQHC (respectively, 14% vs 6%, p=.013; 28% vs 13%, p<.001). In sensitivity analysis of missing data, the most conservative model which imputed missing values as zero saw attenuation of the association between FQHC and the proportion of telehealth delivered via audio-only, as well as between uninsured payer mix > 20% of revenue and telehealth use (p=.16 and p=.09 respectively); all other associations (e.g., rurality, telehealth enablement) retained significance. In the model where missing values were imputed at median telehealth use rates, there was no change in the significance of findings reported in the manuscript.

## References

1. Schifferdecker KE, Yang C-WW, Mackwood MB, Rodriguez HP, Shortell SM, Akré E-R, O’Malley AJ, Butler C, Berube AD, Andrews AO. Safety Net Primary Care Capabilities After the COVID-19 Pandemic. JAMA Health Forum; 2024. p. e242547–e242547.

2. Berg KA, Dalton JE, Gunzler DD, Coulton CJ, Freedman DA, Krieger NI, Dawson NV, Perzynski AT. The ADI-3: a revised neighborhood risk index of the social determinants of health over time and place. Health Services and Outcomes Research Methodology 2021;21(4):486–509.

3. Washington State Department of Health. Guidelines for Using Rural-Urban Classification Systems for Community Health Assessment. 2016. Available from: https://www.doh.wa.gov/Portals/1/Documents/1500/RUCAGuide.pdf [accessed Jan 10, 2024]

4. OneKey Healthcare Industry Database - Overview. IQVIA. Available from: https://www.onekeydata.com/onekey/overview [accessed Jun 13, 2024]

5. Ookla’s Open Data Initiative | Ookla®. Ookla - Providing network intelligence to enable modern connectivity. Available from: https://www.ookla.com/ookla-for-good/open-data [accessed Dec 17, 2024]
